# PI3K inhibitor enhances the cytotoxic response to etoposide and cisplatin in a newly established neuroendocrine cervical carcinoma cell line

**DOI:** 10.18632/oncotarget.17335

**Published:** 2017-04-21

**Authors:** Zih-Yin Lai, Hsin-Yueh Yeo, Ya-Tse Chen, Kuo-Ming Chang, Tze-Chien Chen, Yung-Jen Chuang, Shing-Jyh Chang

**Affiliations:** ^1^ Department of Medical Science and Institute of Bioinformatics and Structural Biology, National Tsing Hua University, Hsinchu 30013, Taiwan (R.O.C.); ^2^ Department of Pathology, Hsinchu MacKay Memorial Hospital, Hsinchu 30071, Taiwan (R.O.C.); ^3^ Department of Obstetrics and Gynecology, MacKay Memorial Hospital, Taipei 10449, Taiwan (R.O.C.); ^4^ Department of Obstetrics and Gynecology, Hsinchu MacKay Memorial Hospital, Hsinchu 30071, Taiwan (R.O.C.)

**Keywords:** neuroendocrine cervical carcinoma, combination therapy, genotoxic drug, PI3K inhibitor, BEZ235

## Abstract

**Background:**

Neuroendocrine cervical carcinoma (NECC) is a rare and aggressive subtype of cervical cancer. To date, no NECC cell-based model is available, which hinders the development of new therapeutic strategies for NECC. In this study, we derived a new NECC cell line from an *ex vivo* biopsy and used it to explore novel drug combination approach for NECC.

**Results:**

The stable HM-1 cell line displayed high expression levels of the neuroendocrine marker, synaptophysin. HM-1 cell transplantation could induce tumor growth in nude mice. As expected, the combination of etoposide and cisplatin synergistically inhibited HM-1 cell proliferation. Strikingly, when etoposide and cisplatin were combined with PI3K inhibitor BEZ235, the growth of HM-1 cells was significantly reduced. Taken together, the data implied the combination of etoposide and cisplatin with BEZ235 not only inhibited HM-1 cell proliferation but also increased cell apoptosis.

**Materials and Methods:**

A NECC tissue sample from a 75-year-old female patient was processed to derive a primary cell line annotated as HM-1. The features of HM-1 were analyzed to establish its characteristic profile. Next, HM-1 was treated with PI3K inhibitors, BKM120 and/or BEZ235, in combination with two well-known genotoxic drugs, etoposide and/or cisplatin, to evaluate which combination could serve as a more effective treatment approach. Their inhibiting effects on HM-1 were evaluated by cell viability, apoptosis, and target kinase expression.

**Conclusions:**

The newly established NECC cell line HM-1 could serve as a cell-based model for NECC research. The synergistic drug combination of PI3K inhibitor with genotoxic drugs might become a potential new treatment strategy against NECC.

## INTRODUCTION

Cervical cancer is one of the leading gynecological cancers contributing to female death worldwide. A recent study has reported 528, 000 new cases of cervical cancer and 266, 000 deaths in 2012 [1]. By histological classification, the most common types of cervical cancer are squamous cell carcinoma (70%) and adenocarcinoma (25%) [2]. In contrast, the neuroendocrine cervical carcinoma (NECC) is a rare subtype of cervical cancer, and accounts for only approximately 2% of all cervical malignancies [3]. Moreover, NECC is aggressive and metastasizes at high rate in clinical observations. The 5-year survival rate for NECC (35.7%) is much lower than squamous cell carcinoma (60.5%) and adenocarcinoma (69.7%) combined [4].

Given the rare prevalence of NECC, there is a limitation in the attempt to determine an optimal treatment. Most published studies contain few patients, and there are no completed prospective trials [5, 6]. Treatment considerations are generally restricted by current options for neuroendocrine carcinoma of the lung [6]. Moreover, the understanding of NECC has been limited by the lack of appropriate cell lines. Therefore, establishing a new NECC cell line shall provide a representative research system to develop therapeutic applications for neuroendocrine cervical carcinoma.

According to the treatment options recommended by the Society of Gynecologic Oncology for neuroendocrine tumors of the gynecologic tract, a combination etoposide and cisplatin chemotherapy should be used for patients at all stages of NECC [7]. These two genotoxic drugs have been commonly used in combination to treat various cancers, including adrenocortical cancer, small cell lung cancer, germ cell tumors, and neuroendocrine tumors [8, 9]. Etoposide is a genotoxic drug with topoisomerase inhibitory activity and works by forming a ternary complex with DNA and the topoisomerase II enzyme, thus preventing re-ligation of the DNA strands and inducing DNA damage [10]. Because cancer cells divide more often than normal cells, etoposide promotes cancer cell apoptosis by causing errors during DNA synthesis [11]. Cisplatin is a platinum-containing genotoxic drug that displays inhibitory activity against a wide variety of solid tumors [12]. Cisplatin works by forming irreversible crosslinks with DNA, which ultimately leads to cell apoptosis. Although the etoposide and cisplatin combination regimen has been recommended for use against NECC, the mortality rate remains high [5]. Therefore, new treatment strategies against this aggressive malignancy are still needed.

Target therapy research has been envisioned to yield effective treatment options for patients who do not respond favorably to standard therapy. Moreover, rare cancers that have limited treatment options can potentially be treated with specific targeted therapies under a precision approach [13]. One example is targeting thePI3K/Akt/mTOR signaling pathway, which plays a critical role in cell growth, differentiation, and apoptosis [14]. Dysregulation of this pathway has been linked to the development of several tumor types and to the pathogenesis of other cancers [15, 16]. In neuroendocrine tumors, nearly all the PI3K/Akt/mTOR pathway components can be molecularly altered, likely with one or more mutations. Thus, this pathway has been listed as a potential drug target for neuroendocrine tumors [17, 18]. Recently, Frumovitz *et al*. found that PIK3CA, which encodes the p110α catalytic subunit of PI3K, is the most common mutation found in small cell neuroendocrine cervical cancer [19]. Other reports also found that the PIK3CA gene is frequently amplified in cervical cancers (68%). PIK3CA mutation is also found in cancers of the endometrium (23%) and ovary (8%) [20, 21]. A recent study showed that small molecule PI3K inhibitors, BKM120 and BEZ235, could effectively reduce cell proliferation and increase apoptosis in pancreatic neuroendocrine tumor cells [22]. Therefore, we investigated whether these two PI3K inhibitors with/without genotoxic agents may display anti-NECC efficacy and explored the feasibility of using the drugs in combination as new treatment options for NECC patients.

In this study, we first established a new NECC cell line to overcome the lack of a research model. This NECC cell line was then used to investigate the pathological characteristics of NECC for determining potential treatment strategies. Combination treatment with genotoxic drugs (etoposide and cisplatin) and a PI3K inhibitor was evaluated for NECC cell growth inhibition and apoptosis.

## RESULTS

### General characteristics of HM-1 cells derived from human NECC: morphology, growth rate and HPV infection

**Figure 1:**
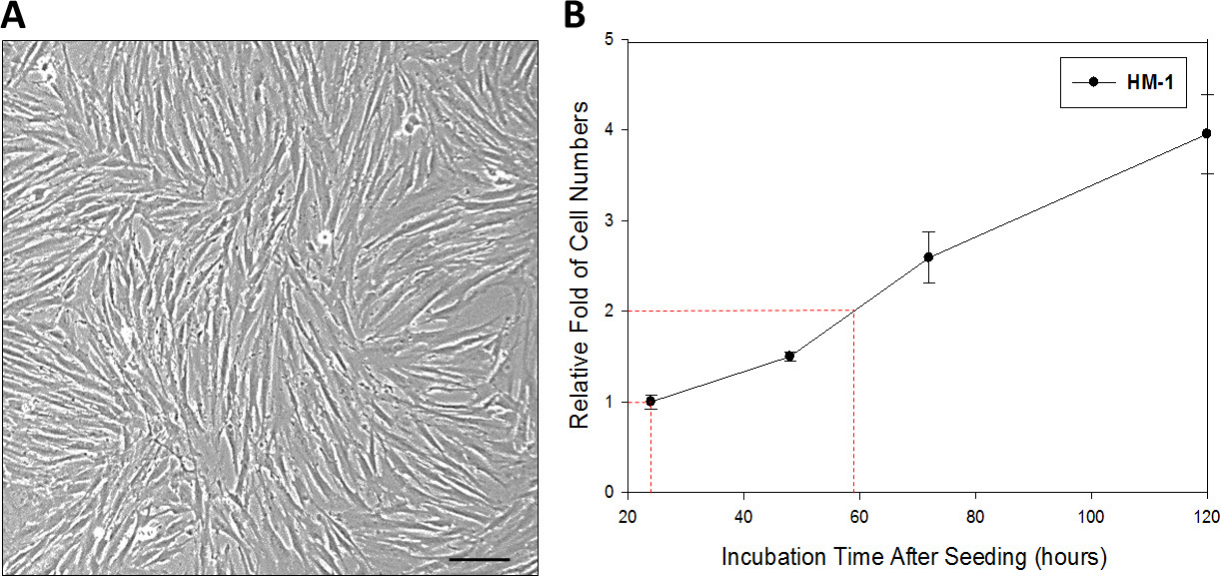
Characterization of HM-1 cells (**A**) Morphological presentation of HM-1 cell culture. (Scale bar represented 100 μm) (**B**) Cell growth curve assay was performed in 6-cm petri dish; 1 × 10^5^ HM-1 cells were seeded and counted every 24 hours. The HM-1 cell population was estimated to double every 35 hours. The data represented the mean ± SEM.

Cellular morphology of HM-1 was examined under a phase contrast microscope (Figure 1A). HM-1 cells were either bipolar or multipolar and displayed a fibroblast-like shape in culture. After reaching confluence, HM-1 had an elongated, spindle shape. Furthermore, HM-1 had the tendency to lose contact inhibition upon prolonged culturing.

We next investigated the growth rate of HM-1 cells by cell doubling time assay (Figure 1B). After seeding 1 × 10^5^ HM-1 cells in 6-cm petri dishes, the cells were maintained and harvested every 24 hours for cell counting. The result showed that the doubling time of HM-1 cells was 35 hours *in vitro*. By using RT-PCR assay to detect HPV infection in HM-1 cells (Supplementary Figure 1), we found HM-1 expressed a positive match of HPV-16 infection. This result suggested a past HPV-16 infection in HM-1. Authentication of HM-1 cells by STR DNA Profiling was also performed (Supplementary Table 1) and used for comparison and to check the identity against other cell lines in the STR reference database (data not shown).

### HM-1 cells expressed the neuroendocrine marker synaptophysin (SYP) and expanded *in vivo* via xenotransplantation

**Figure 2:**
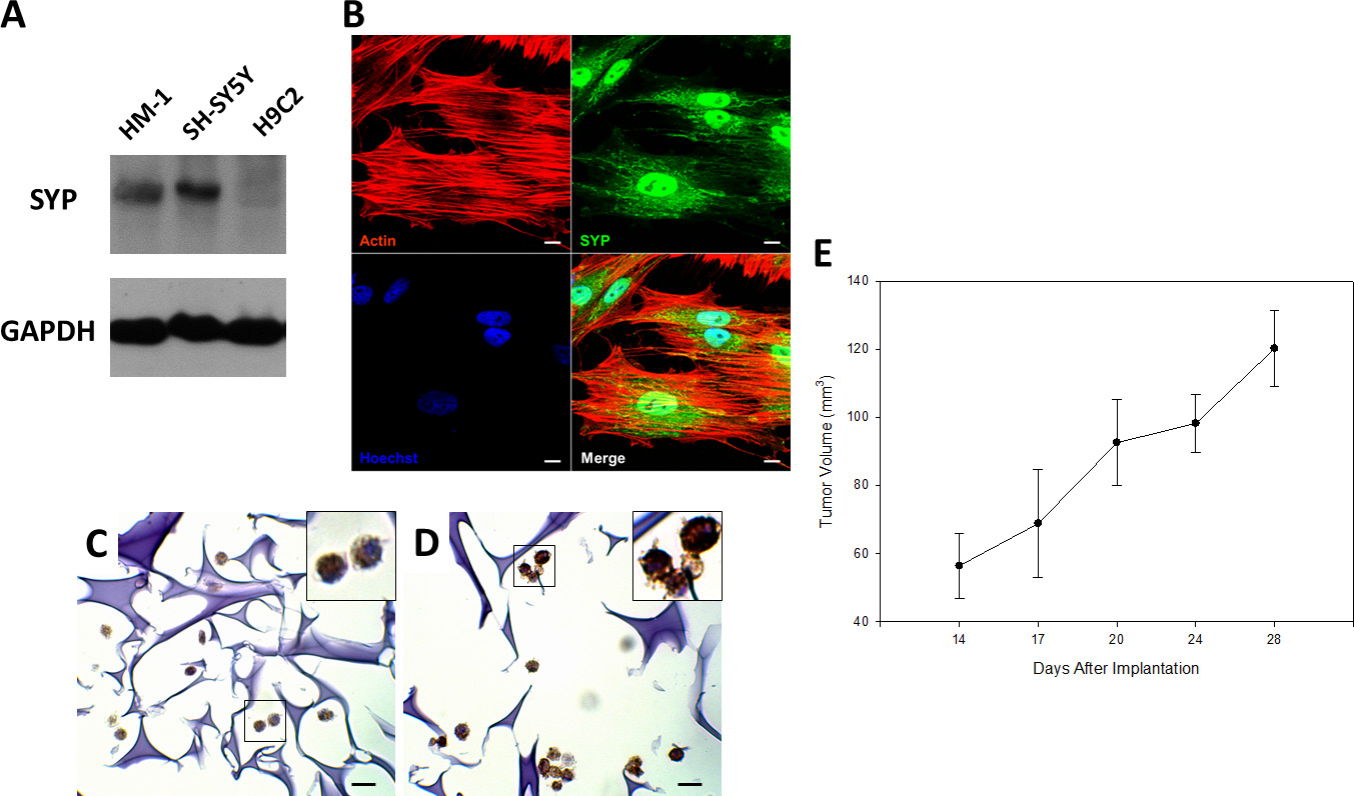
HM-1 cells expressed the neuroendocrine marker neuroendocrine synaptophysin (SYP) and xenotransplantation (**A**) Western blot analysis on cell lysates showed HM-1 cells and the positive control human neuroblastoma SH-SY5Y cells both expressed SYP. H9C2 (rat derived cardiac myoblast cell line) was the negative control, while GAPDH was the loading control. (**B**) Confocal image further demonstrated SYP protein was expressed in HM-1 cells, the pattern supported SYP's abundant presence in the vesicles. Anti-SYP staining (green) was shown in the upper-right panel; actin (red) and nucleus (blue by Hoechst stain) was shown as anatomical landmarks. (Scale bar represented 10 μm) (**C**) Cell block staining re-confirmed that HM-1 cells was positively stained for SYP. The upper right image was enlarged view of the black boxed region. (Scale bar represented 10 μm) (**D**) Cell block staining also showed that HM-1 cells strongly expressed the neural cell adhesion molecule protein (CD56) to verify its neural cell origin. The upper-right inner image was enlarged view of the boxed region. (Scale bar represented 10 μm) (**E**) 5 × 10^6^ HM-1 cells were subcutaneously inoculated into the back of BALB/c female nude mice to track its growth ability *in vivo*. Tumor size increased continuously after inoculation, and the tumor volume was calculated by the following equation: width^2^ × length × 1/2 (in mm^3^). The data represented the mean ± SEM.

We next determined whether HM-1 expressed the well-known neuroendocrine marker, synaptophysin (SYP) [6, 23] by Western blot and immunocytochemistry assay (Figure 2A–2B). In Western blot analysis, we used the neuroblastoma cell line SH-SY5Y and the cardiac myoblast cell line H9C2 as the positive and negative controls, respectively. The result indicated that HM-1 cells evidently expressed SYP. The immunocytochemistry assay likewise confirmed the SYP expression in HM-1 cells, and the expression pattern supported the abundant presence of SYP in the vesicles. Cell block staining also showed that HM-1 cells positively expressed SYP (Figure 2C) and the neural cell adhesion molecule protein CD56 protein (Figure 2D). Taken together, these data validated the neuroendocrine lineage of HM-1 cells.

To investigate the *in vivo* tumorigenicity of HM-1 cells, we inoculated 5 × 10^6^ HM-1 cells subcutaneously into the back of BALB/c female nude mice and monitored the growth of tumors. A growth curve of HM-1 showed the tumor volume increased progressively during the first month after transplantation (Figure 2E). We estimated the *in vivo* tumor volume doubling time of HM-1 to be approximately 13 days. These findings demonstrated that HM-1 was a neuroendocrine tumor with carcinogenicity *in vivo*.

### Combination of etoposide and cisplatin effectively inhibited the growth of NECC

**Figure 3:**
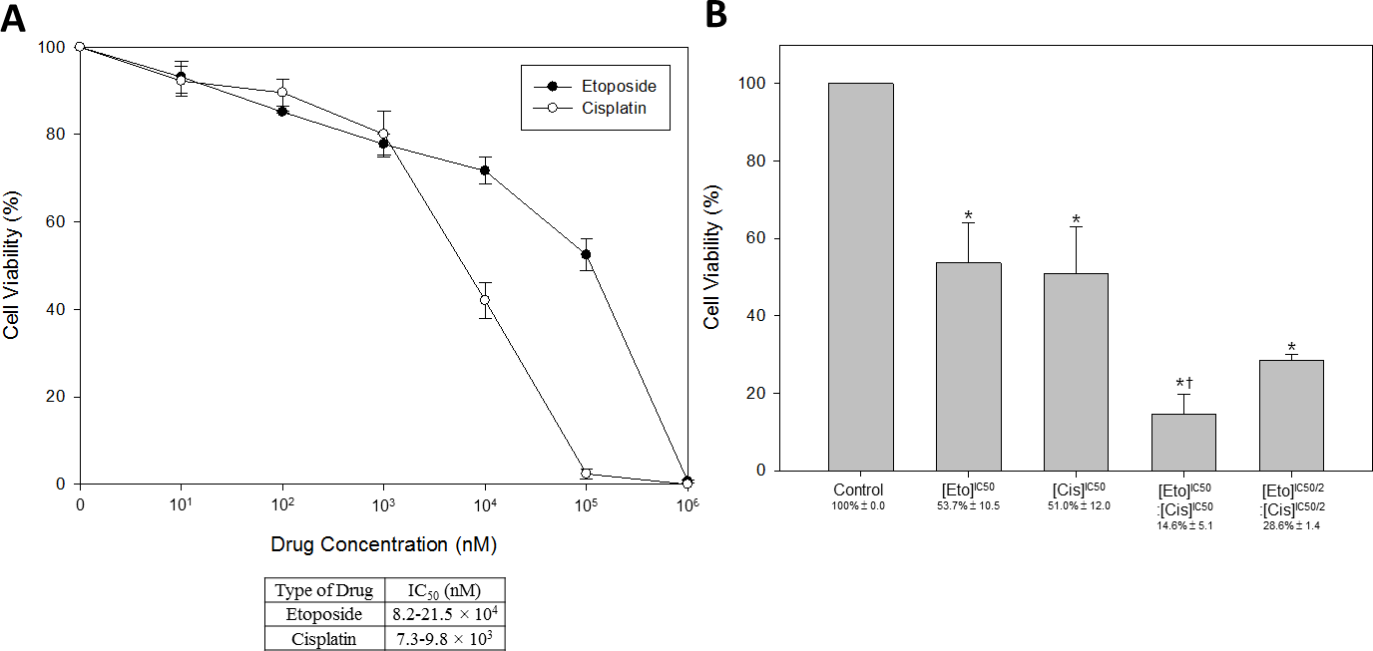
Dose response curves of HM-1 cells to etoposide and cisplatin treatment Two of the most common chemotherapeutic drugs for NECC were tested: etoposide (a topoisomerase inhibitor) and cisplatin (an alkylating agent), by cell viability assay. (**A**) The 50% growth-inhibitory concentration (IC_50_, nM) to each drug was 8.2–21.5 × 10^4^ nM for etoposide, and 7.3–9.8 × 10^3^ nM for cisplatin. (**B**) The anti-cancer efficacy of etoposide and cisplatin (EP) in combination on HM-1 was significantly increased. The controls were untreated cells, etoposide alone, and cisplatin alone. The etoposide and cisplatin combination with 1-to-1 ratio at IC_50_ performed better than each drug alone and resulted in a cell viability of 14.6% as compared to the untreated control. Reducing the concentrations of both drugs by half could still result in a cell survival rate of 28.6% comparted to the untreated control (*p < 0.05 vs. control; ^†^*p* < 0.05 vs. single-drug treatment). The data represented the mean ± SEM.

To evaluate the sensitivity of HM-1 to the current NECC chemotherapy drugs, two FDA approved agents, etoposide (topoisomerase II inhibitor) and cisplatin (alkylating agent), were selected for this experiment. The drug concentrations ranged from 0 to 10^6^ nM, and the exposure time was 72 hours (Figure 3A). Due to the 50% growth-inhibitory (IC_50_) variability of etoposide and cisplatin in each repeat at different drug concentrations, the range of IC_50_ values on HM-1 were selected to be 8.2–21.5 × 10^4^ nM for etoposide, and 7.3–9.8 × 10^3^ nM for cisplatin. We found both agents could effectively reduce HM-1 cell viability at concentrations higher than 10^3^ nM. Moreover, the data suggested that cisplatin had a higher inhibition efficacy on HM-1 cell viability.

According to the treatment options recommended by the Society of Gynecologic Oncology for neuroendocrine tumors of the gynecologic tract, a combination chemotherapy of etoposide and cisplatin should be used for patients at all stages of NECC [7]. Therefore, we investigated the combined anti-cancer efficacy of etoposide and cisplatin (EP) on HM-1 (Figure 3B). The experimental HM-1 cell group was treated for 72 hours with a combination of etoposide and cisplatin, using a 1-to-1 ratio at their IC_50_ concentrations (calculated from data shown in Figure 3A). The results showed this combination had a much stronger inhibitory effect on HM-1 cells. Specifically, the double-drug treatment reduced cell viability compared to the single-drug treatments from 51%∼53.7% to 14.6%. To verify the synergistic effect of the etoposide and cisplatin combination, we also performed another set of assays at the halved IC_50_ (IC_50/2_) condition. We found even though the co-treatment concentration was reduced by half, the cell viability could still be dropped to 28.6% compared with single-drug treatments. Consistent with previous clinical reports, these results indicated that the etoposide and cisplatin combination was effective in killing HM-1 *in vitro* and supported the application of such a drug combination for treating NECC.

### PI3K inhibition decreased HM-1 cell proliferation

**Figure 4:**
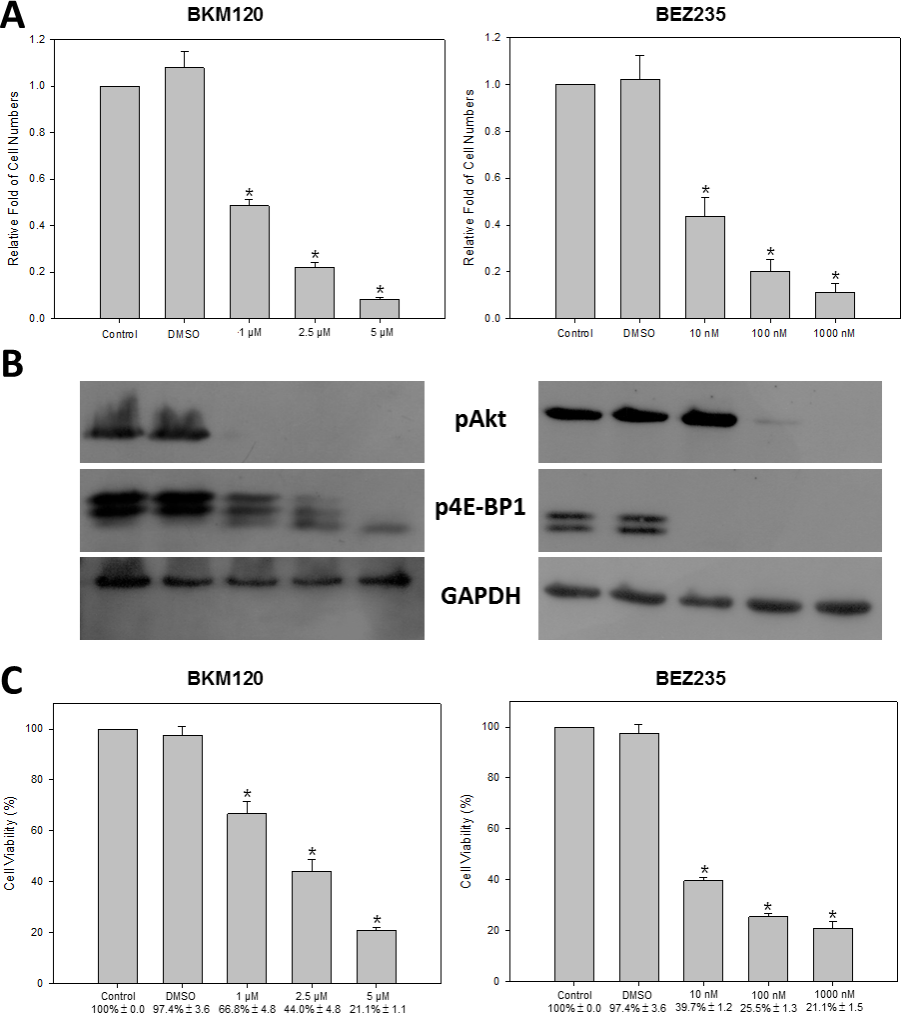
PI3K inhibitors decreased cell proliferation rate and cell viability of HM-1 cells HM-1 cells were seeded in culture plates and treated with various doses of BKM120 (1, 2.5, or 5 μM) or BEZ235 (10, 100, or 1000 nM). (**A**) The cell numbers were significantly decreased at all doses under either BKM120 or BEZ235 treatment (*p < 0.05 vs. control). (**B**) The Western blot analysis showed the levels of PI3K signaling downstream proteins, pAkt and p4E-BP1, were significantly reduced with increasing concentrations of either inhibitor. GAPDH was the loading control. (**C**) The cell viability (%) determined by MTT/WST-1 assay was significantly reduced at all concentrations for either BKM120 or BEZ235 (*p < 0.05 vs. control). The data represented the mean ± SEM.

To explore alternative drugs and drug combinations that could be more effective for treating NECC, we evaluated the anti-cancer efficacy of PI3K inhibitors on HM-1. HM-1 cells were treated with two PI3K inhibitors: BKM120 and BEZ235 at different concentrations. Cell numbers were determined at 72 hours after each treatment (Figure 4A). We found HM-1 cell numbers were significantly reduced at all doses in the BKM120 and BEZ235 groups compared with the no-treatment control and DMSO groups. To determine whether the PI3K downstream signaling pathway was suppressed by BKM120 and BEZ235, we harvested the HM-1 lysates and analyzed the status of Akt and 4E-BP1 phosphorylation by Western blot (Figure 4B). We found that with increasing doses of BKM120, the phosphorylation level of Akt gradually decreased. Similarly, treatment with increasing concentrations of BEZ235 also reduced the phosphorylated Akt level but at a much lower concentration of inhibitor. In addition, the phosphorylated form of another downstream regulatory protein 4E-BP1 showed a dose-dependent reduction under BKM120 treatment. On the other hand, the phosphorylated 4E-BP1 was completely eliminated in the presence of BEZ235, which suggested that BEZ235 caused an effective down-regulation of p4E-BP1 in HM-1. We also performed viability assay to examine the effects of PI3K inhibitors on HM-1 (Figure 4C). As expected, treatment with various concentrations of BKM120 and BEZ235 demonstrated a dose-dependent inhibition of HM-1 cell viability.

### PI3K inhibition resulted in an increase in HM-1 apoptosis and DNA damage

**Figure 5:**
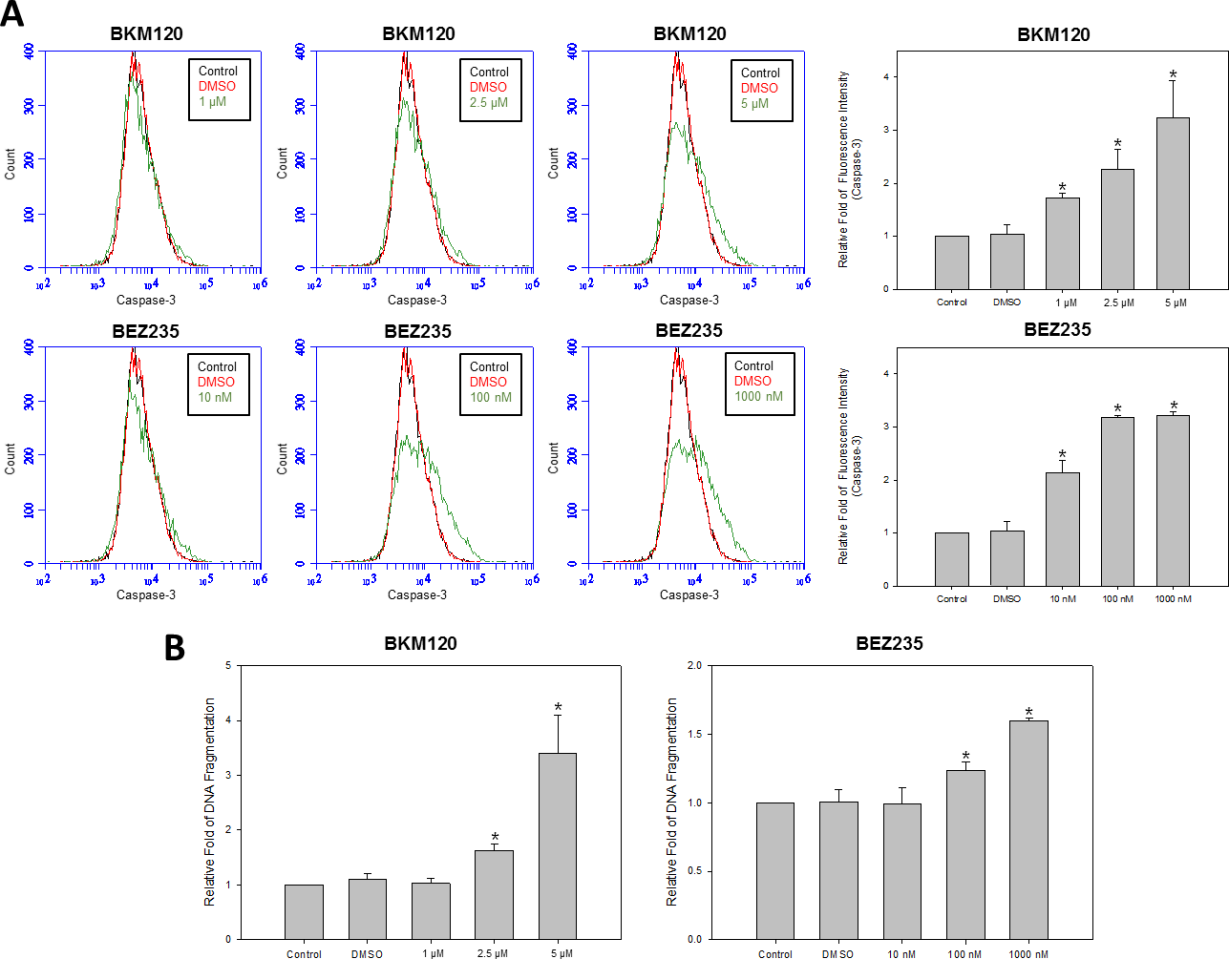
PI3K inhibitors enhanced cell apoptosis of HM-1 cells HM-1 cells were seeded in plates and treated with various concentrations of BKM120 (1, 2.5, or 5 μM) or BEZ235 (10, 100, or 1000 nM). (**A**) HM-1 cells were pre-processed with PE active caspase-3 apoptosis kit (BD Bioscience) before flow cytometry analysis. Top panels were flow cytometry histograms of HM-1 cells, which were incubated with control (black), DMSO (red), or different concentration of BKM120 or BEZ235 (green). Bottom panels were the quantitative bar graphs to represent the normalized statistical data of caspase-3 activity in the histograms above. Under all concentrations of BKM120 and BEZ235 treatment, events of cell apoptosis were significantly increased (*p < 0.05 vs. control). (**B**) DNA fragmentation was significantly increased at ≥ 2.5 μM of BKM120 and ≥ 100 nM of BEZ235. DNA fragmentation was measured using the Cell Death Detection ELISA^plus^ Kit (*p < 0.05 vs. control). The data represented the mean ± SEM.

Because PI3K signaling is known to regulate cell growth and survival, we evaluated whether the PI3K inhibitors mechanisms of action toward HM-1 was associated with cell apoptosis and DNA damage. For this experiment, we performed apoptotic marker caspase-3 activation analysis by flow cytometry (Figure 5A). After treating HM-1 with various doses of BKM120 and BEZ235, the mean values of caspase-3 fluorescence intensity were significantly increased as shown by the widening base of signals moving toward the right. Furthermore, the quantitative and statistical illustrations of the cytometric histograms showed each PI3K inhibitor had a dose-dependent increase of activated caspase-3 compared with the control and DMSO groups. To further analyze this apoptotic effect, HM-1 cells were treated with each PI3K inhibitor at three different concentrations for 24 hours before DNA fragmentation analysis (Figure 5B). Consistent with earlier findings, DNA fragmentation was effectively induced by ≥ 2.5 μM of BKM120 or ≥ 100 nM of BEZ235.

### The triple-drug combination of etoposide, cisplatin and BEZ235 was the most effective in suppressing HM-1 viability and increasing HM-1 apoptosis *in vitro*

**Figure 6:**
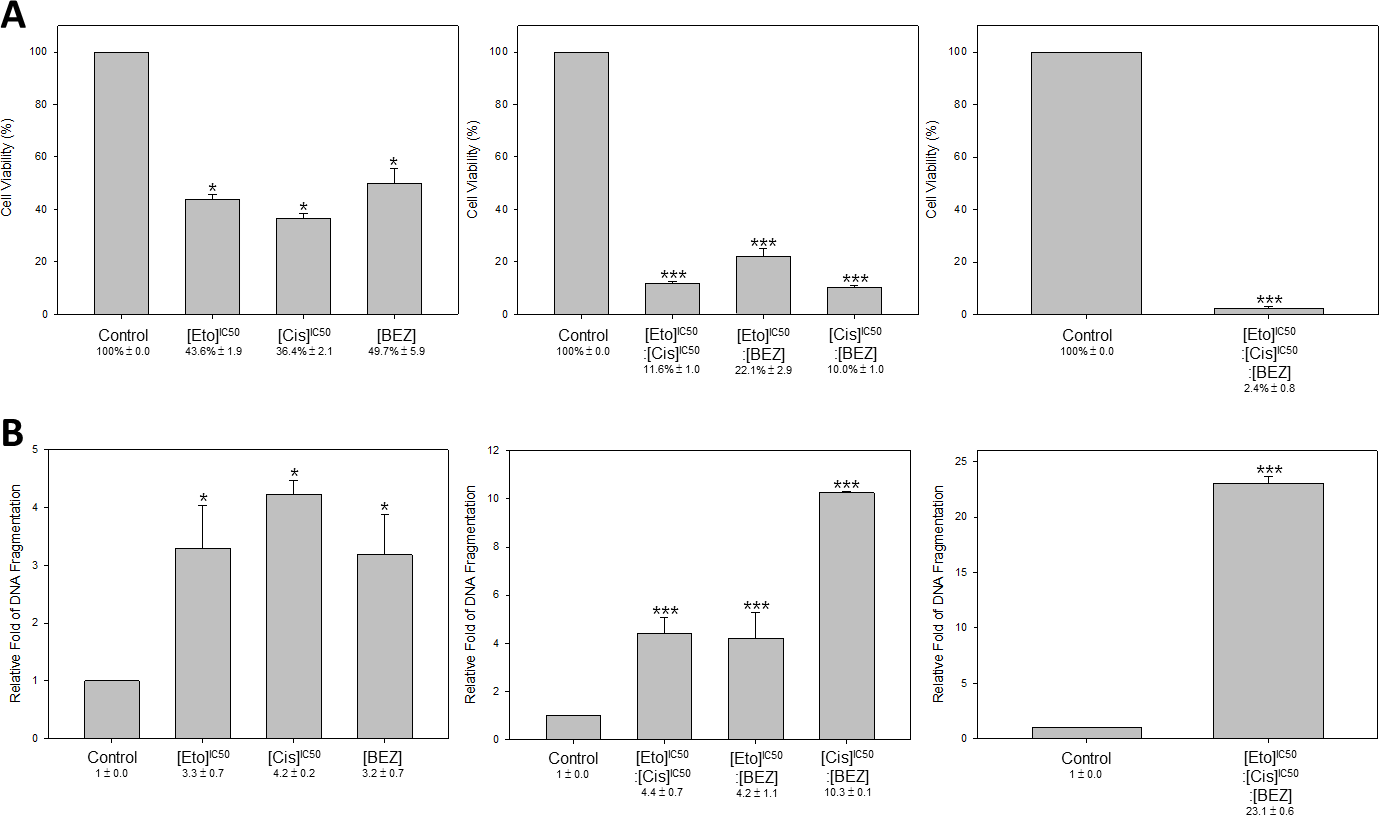
Triple-drug combination of etoposide, cisplatin and BEZ235 resulted in more effective cell inhibition and DNA damage in HM-1 HM-1 cells were seeded in plates and were either non-treated control or treated with etoposide (8.2–21.5 × 10^4^ nM), cisplatin (7.3–9.8 × 10^3^ nM), BEZ235 (10 nM), or combination of etoposide + cisplatin (8.2–21.5 × 10^4^ nM + 7.3–9.8 × 10^3^ nM), etoposide + BEZ235 (8.2–21.5 × 10^4^ nM + 10 nM), cisplatin + BEZ235 (7.3–9.8 × 10^3^ nM + 10 nM), etoposide + cisplatin + BEZ235 (8.2–21.5 × 10^4^ nM + 7.3–9.8 × 10^3^ nM + 10 nM). (**A)** The single- or double-drug treatments reduced HM-1 cells viability as expected. Notably, the cell viability (%) was significantly down-regulated in the triple-drug combination group (*p < 0.05 vs. control; ***p < 0.001 vs. control). (**B**) DNA fragmentation was significantly increased at all treatment groups, especially in the triple-drug (etoposide, cisplatin and BEZ235) combination group. The DNA fragmentation was determined using the Cell Death Detection ELISA^plus^ Kit (*p < 0.05 vs. control; ***p < 0.001 vs. control). The data represented the mean ± SEM.

After confirming PI3K inhibitors were effective anti-HM-1 agents, we next determined which drug combination would have an anti-cancer response better than the etoposide and cisplatin (EP) combination. Because it was evident that HM-1 was more sensitive to BEZ235, we devised the following treatment options for anti-HM-1 drug combination screening: three single-drug treatments including etoposide only, cisplatin only, and BEZ235 only; three double-drug combinations including “etoposide + cisplatin”, “etoposide + BEZ235”, “cisplatin + BEZ235”; and “etoposide + cisplatin + BEZ235”. The cell viability assay was examined at 72 hours after each treatment (Figure 6A).

We found all treatment options could inhibit HM-1 cell viability. For single-drug treatments, the cell viability rates were dropped to 36.4%∼49.7%. The anti-cancer effects could be further enhanced under the double-drug combinations to the level of 10.0% to 22.1%. Interestingly, HM-1 viability was significantly reduced under the “etoposide + cisplatin + BEZ235” triple-drug treatment group, which resulted in a striking 2.4% cell viability compared to the control.

We next measured the extent of DNA damage in HM-1 in response to each treatment condition (Figure 6B). The result showed that “etoposide + cisplatin” increased the DNA fragmentation compared with either etoposide or cisplatin alone or control group. Notably, the triple-drug combination of “etoposide + cisplatin + BEZ235” resulted in an even greater inhibition efficacy of HM-1, which was demonstrated by the 23-fold enhancement compared with the control group alone. These data implied the potential of adapting a triple-drug combination to develop a novel therapeutic strategy for NECC.

## DISCUSSION

Limited by the rare occurrence of NECC, previous studies have suggested that the establishment of neuroendocrine tumor cell lines may facilitate the understanding of neuroendocrine tumor biology and the development of new therapeutic modalities [24, 25]. Hence, developing a representative NECC cell line is important for advancing NECC research. In this study, we successfully established a neuroendocrine cervical carcinoma (NECC) cell line, HM-1, from a 75-year-old female. This cell line was characterized by neuroendocrine marker expression and carcinogenic properties. Further analysis validated HM-1 as a novel, stable and tumorigenic cell line that possesses the crucial characteristics of neuroendocrine tumors and has the potential to serve as a cell-based NECC research model.

To date, the combined etoposide and cisplatin genotoxic regimen is a widely accepted chemotherapy approach for treating NECC [7, 26–28]. In accordance with this recommendation, our data also supported that a combination of etoposide and cisplatin could synergistically inhibit HM-1 cells compared with the individual agents alone (Figure 3). To enhance the anti-cancer efficacy, we further explored new therapeutic modalities for NECC. We hypothesized that by co-targeting the PI3K signaling transduction pathway, we might observe better anti-cancer efficacy than the current genotoxic drug combination [15]. We henceforth applied and found that PI3K inhibitors BKM120 and BEZ235 could significantly inhibit HM-1 cell proliferation (Figure 4) and induce cell apoptosis (Figure 5). These results indicated that the PI3K/Akt/mTOR signaling pathway might be involved in driving NECC progression.

The pan-PI3K inhibitor BKM120 has been shown to have anti-cancer activity in preclinical models of various solid tumors [29–31]. While favorable tolerability profiles with common adverse events have been observed, BKM120 displayed modest clinical efficacy under a single-agent regimen in early clinical trials [32]. BEZ235 is a PI3K and mTOR dual-specificity inhibitor that simultaneously targets the ATP binding sites of PI3K and mTOR [33]. BEZ235 has been shown to possess favorable pharmaceutical properties in preclinical studies [34, 35]. Strikingly, we noticed that BEZ235 could effectively kill HM-1 cells at a much lower concentration than that of BKM120. Such variation in chemo-sensitivity has also been observed in breast cancer cells [36], lymphoma cells [37] and leiomyosarcoma cells [38]. Thus, BEZ235 was selected for subsequent assessments in a drug combination screen.

As mentioned above, multimodality therapy of etoposide/cisplatin is the recommended approach for treating NECC [28]. Based on this, we analyzed the combined effects of etoposide/BEZ235 and cisplatin/BEZ235 for their anti-HM-1 activities. We found that the combination of cisplatin and BEZ235 shared similar inhibitory effects on HM-1 viability with the combination of etoposide/cisplatin. Interestingly, the combination of cisplatin and BEZ235 displayed a higher synergistic effect, which was associated with a two-fold increase of HM-1 cell DNA fragmentation compared with the combination of etoposide and cisplatin (Figure 6). However, the combination of etoposide and BEZ235 presented a weaker anti-HM-1 activity than the combination of etoposide/cisplatin. Together, our data indicated that BEZ235 in combination with cisplatin could induce a stronger cytotoxic response in HM-1 than the recommended etoposide/cisplatin combination used in current NECC therapy. In agreement with our findings, a previous report also showed that the combination of cisplatin and BEZ235 had a better inhibitory effect on medulloblastoma than the combination of etoposide and BEZ235 [39]. These observations imply we might anticipate an improved efficacy of cisplatin plus BEZ235 for treating NECC patients. Our data demonstrated that HM-1 cell viability could be further inhibited under the triple-drug combination, in which BEZ235 plus etoposide and cisplatin generated the highest inhibitory effect (Figure 6). Notably, this triple-drug combination also significantly induced HM-1 cell DNA fragmentation. Thus, genotoxic agents plus PI3K inhibitor could be a novel and effective therapeutic option for treating NECC. Our data could serve as pre-clinical evidence for initiating trials combining PI3K inhibitors with the current etoposide/cisplatin regimen for NECC patients.

We acknowledged that the HM-1 cell line was derived from only one NECC patient. Nonetheless, due to its rare occurrence, no other NECC cell lines were available to be included in this study [40]. Moreover, the drug sensitivity of neuroendocrine tumor cell lines showed wide variations in recent reports [41, 42]. Thus, we recognized that more clinical samples should be collected to explore the NECC tumor progression process and to verify the drug mechanism of action before launching a clinical trial to determining the optimal NECC multi-drug regimens.

**Figure 7:**
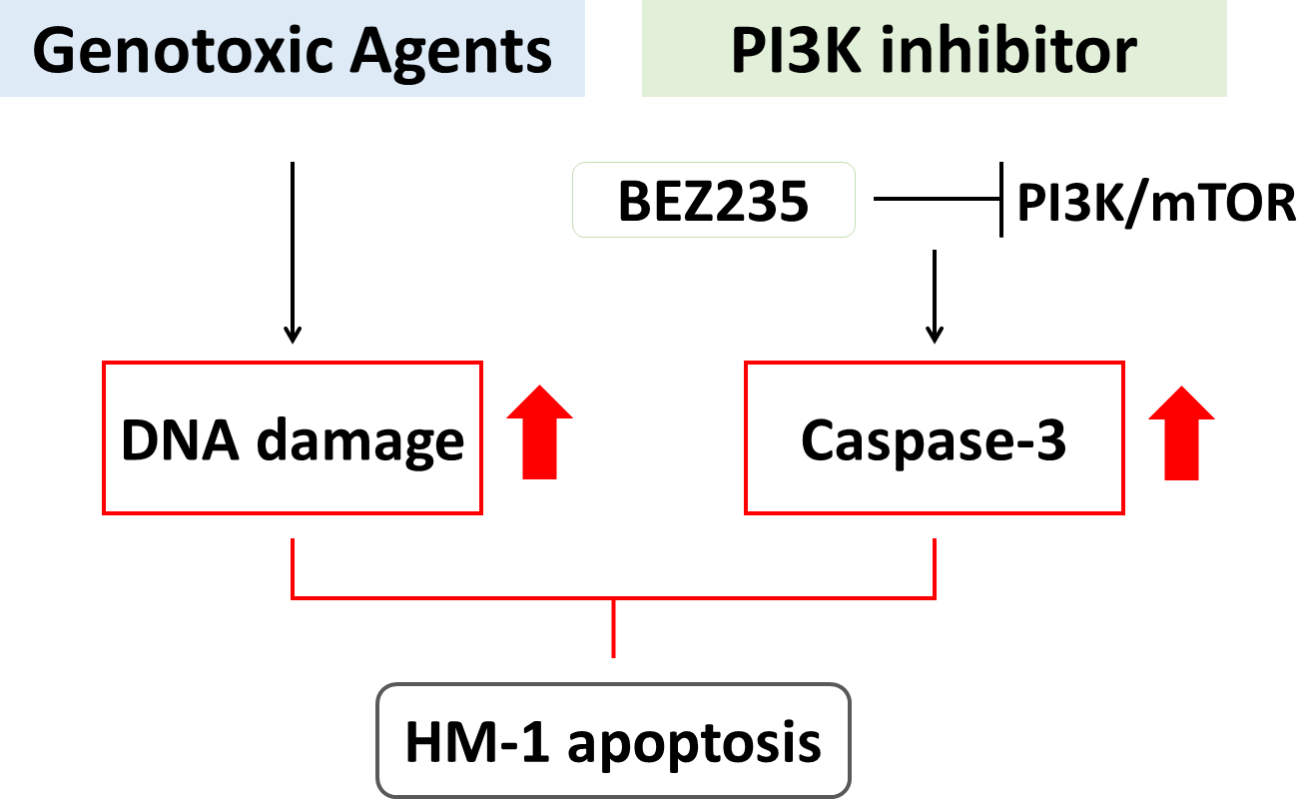
Schematic model for a novel therapeutic strategy for treating NECC Genotoxic agents (i.e., etoposide and cisplatin) could induce DNA damage, while PI3K inhibitor (i.e., BEZ235) blocked PI3K/mTOR pathway and induced caspase-3 activity. With the combination of genotoxic agents and BEZ235, the synergistic effect could further up-regulate the HM-1 apoptosis. The drug combination of genotoxic agents and BEZ235 might be a novel therapeutic option for NECC patients.

In summary, we showed that the new NECC cell line HM-1 could be a representative cell-based model for NECC research. Our data demonstrated that combination treatment with the genotoxic drugs etoposide and cisplatin with BEZ235 resulted in enhanced cell cytotoxic responses in HM-1 by reducing cell viability and increasing cell apoptosis. Our results suggested that the triple-drug combination of genotoxic drugs with PI3K inhibitor present a potential therapeutic approach against NECC (Figure 7). We predict that this novel human tumor-derived cell line will be an important model system for studying NECC tumorigenesis. By screening potential anticancer agents and discovering predictive biomarkers, further analysis on HM-1 might provide insights for the development of new NECC treatment strategies.

## MATERIALS AND METHODS

### Ethics approval and consent to participate

This study was approved by the institutional review boards of the MacKay Memorial Hospital (IRB Approval No. 12MMHIS082). The only participant had provided written informed consent. The protocols of this study were consistent with the ethical guidelines of the 1975 Helsinki Declaration.

### Cancer cell isolation and cell culture

In this study, we obtained the valuable neuroendocrine cervical carcinoma tissue sample from a 75-year-old female patient by the Cancer Cell Isolation Kit (Panomics), following the manufacturer's instruction, to isolate the primary cells. The dissociated cells were then cultured on flasks in RPMI-1640 culture medium (Gibco) containing 10% heat-inactivated fetal bovine serum, 100 units/mL penicillin, 100 μg/mL streptomycin and 2.5 μg/mL of amphotericin B at 37°C with 5% CO_2_. Colony derived from single cell was then selected and transferred to 96-well plates for continued culturing. We noted that HM-1 cells required a minimum cell density (3 × 10^3^ cells/cm^2^) to grow. This primary cultured cancer cell line was annotated as HM-1 (Hsinchu MacKay-1).

### Doubling time assay

A total of 1 × 10^5^ HM-1 cells were seeded in 6-cm petri dishes and cultured at 37°C in ambient air with 5% CO_2_ for 24 hours. The cells were cultured and harvested every 24 hours using trypsin-EDTA (Gibco). Then, cells were counted by hemocytometer. We calculated the doubling time as follows: 59 (relative fold of cell numbers = 2) – 24 (relative fold of cell numbers = 1) = 35 hours.

### Short tandem repeat analysis

HM-1 cells were counted and diluted to 1 × 10^6^ cells/mL with PBS. Then, cells were centrifuged again and the supernatant was discarded. Short tandem repeat (STR) analysis was entrusted to GeneLabs Life Science (Taipei, Taiwan). The DNA was amplified using the GenePrint^®^ 10 System, and the STR results were analyzed using the Applied Biosystems 3730 DNA Analyzer and GeneMapper^TM^ Software. The data was shown in the Supplementary Table 1.

### HPV detection

Total RNA from the HM-1 cells was extracted by TRIzol reagent (Invitrogen) according to the manufacturer's protocol. Three micrograms of total RNA were reverse-transcribed using SuperScript^™^ III Reverse Transcriptase (Invitrogen). PCR was performed with Phire Hot Start II DNA Polymerase (Thermo Scientific). The primer sequences were the following: HPV-16, 5′-GCACCAAAAGAGAACTGCAA-3′ and 5′-ATGCATAAATCCCGAAAAGC-3′; HPV-18, 5′-T GTATGGAGACACATTGGAAAA-3′ and 5′-GAGTC GTTCCTGTCGTGCTC-3′; GAPDH, 5′-ACACCC ACTCCTCCACCTTT-3′ and 5′-TACTCCTTGGAGGC CATGTG-3′.

### Western blot analysis

Cell extracts were isolated using RIPA lysis buffer (Roche) and quantitated by BCA protein assay kit (Pierce). Proteins were separated using 10% sodium dodecyl sulfate polyacrylamide gels (SDS-PAGE), and then the gel was transferred onto PVDF membranes (Millipore) using a Tank Transfer System (Bio-Rad). The membranes were then incubated with different primary antibodies overnight at 4°C. Species-specific Horseradish Peroxidase (HRP)-conjugated secondary antibodies were used for detecting the primary antibodies. Detection was accomplished using an ECL chemiluminescence system (GE Healthcare) and the images were exposed to films. The anti-synaptophysin (SYP), pAkt, and p4E-BP1 antibodies were obtained from Cell Signaling Technology. The anti-GAPDH antibody was from Santa Cruz Biotechnology.

### Immunocytochemistry

HM-1 cells were seeded on sterile glass coverslips overnight. Cells were fixed with 4% paraformaldehyde/PBS and permeabilized with 0.1% Triton X-100/PBS. Then, cells were blocked with 3% BSA/PBS at room temperature. After blocking, cells were incubated in blocking solution along with anti-SYP antibody at 4°C overnight. The secondary antibody used was anti-rabbit IgG-DyLight488 (Jackson). Nuclei were stained with Hoechst 33342 (Invitrogen). Phalloidin (Invitrogen) was used to label the actin cytoskeleton. All images were captured using a confocal microscope (LSM510 Meta, Zeiss).

### Xenotransplantation

A total of 5 × 10^6^ HM-1 cells were implanted subcutaneously on the lateral body wall in the axillary region caudal to the foreleg of female BALB/c nude mice aged 6–8 weeks from National Laboratory Animal Center (NLAC) as instructed by the animal center technician. We measured the HM-1 tumor transplanted on the backs using a caliper in two dimensions twice per week, starting 1 week after inoculation, and the tumor volume was calculated (width^2^ × length × 1/2). The mice were sacrificed 4 weeks after cell inoculation, and then tumors were dissected for further histological analysis.

### Genotoxic agents and PI3K inhibitors

Etoposide and cisplatin were purchased from Fresenius Kabi. BKM120 was obtained from Cayman Chemical. BEZ235 was obtained from BioVision.

### Cell proliferation assay

HM-1 cells were seeded in 24-well plates at a density of 3 × 10^4^ cells/well. After 24 hours, drugs were added to each well and incubation continued for 72 hours. Cell proliferation was measured by counting cell numbers using a flow cytometer system (BD Accuri C6). Each experiment was performed in triplicate and repeated at least three times.

### Cell viability assay

HM-1 cells were seeded in 96-well plates at a density of 5 × 10^3^ cells/well. After 24 hours, drugs were added to each well and incubation continued for 72 hours. Cell viability was assessed by MTT (Sigma-Aldrich)/WST-1 (Roche Applied Science) reagent. Following the manufacturer's protocols, 100 μl/well culture medium and 10 μl/well MTT or WST-1 solution were added to 96-well plates and incubated at 37°C and 5% CO_2_ for 4 or 2 hours. Finally, absorbance was measured at 570/450 nm. Each experiment was performed in triplicate and repeated at least three times.

### DNA fragmentation ELISA

HM-1 cells were seeded in 24-well plates at a density of 6 × 10^4^ cells/well. After allowing the cells to adhere, drugs were added to each well for 24 hours. Apoptosis was assessed by DNA fragmentation using the Cell Death Detection ELISA^PLUS^ kit (Roche Applied Science) following the manufacturer's protocol. Each experiment was performed in triplicate and repeated at least three times.

### Cell Caspase-3 apoptosis

HM-1 cells were seeded in 6-well plates at a density of 3 × 10^5^ cells/well. After allowing the cells to adhere, drugs were added to each well for 24 hours. After washing, cells were stained using the PE Active Caspase-3 Apoptosis Kit (BD Biosciences) according to the manufacturer's protocol and analyzed using a flow cytometer system (BD Accuri C6). Each experiment was performed in triplicate and repeated at least three times.

### Statistics

The data were analyzed using SigmaPlot v.10 and expressed as the mean with the standard error of the mean (SEM). Statistical significance was determined by a *p value* < 0.05 using Student's *t-test*.

## SUPPLEMENTARY FIGURE AND TABLE



## Abbreviations

NECC: Neuroendocrine cervical carcinoma
HM-1: Hsinchu MacKay-1
STR: Short tandem repeat
SYP: Synaptophysin
EP: Etoposide and cisplatin
IC50: 50% growth-inhibitory concentration
