# Supplementary material for: PI3K inhibitor enhances the cytotoxic response to etoposide and cisplatin in a newly established neuroendocrine cervical carcinoma cell line
Source: Oncotarget. 2017 Apr 21;8(28):45323–34. doi: 10.18632/oncotarget.17335 (PMC5542189; doi:10.18632/oncotarget.17335)
Supplement: Supplementary file 1 [file oncotarget-08-45323-s001.pdf]

PI3K inhibitor enhances the cytotoxic response to etoposide and cisplatin in a newly established neuroendocrine cervical carcinoma cell line

Supplementary Materials

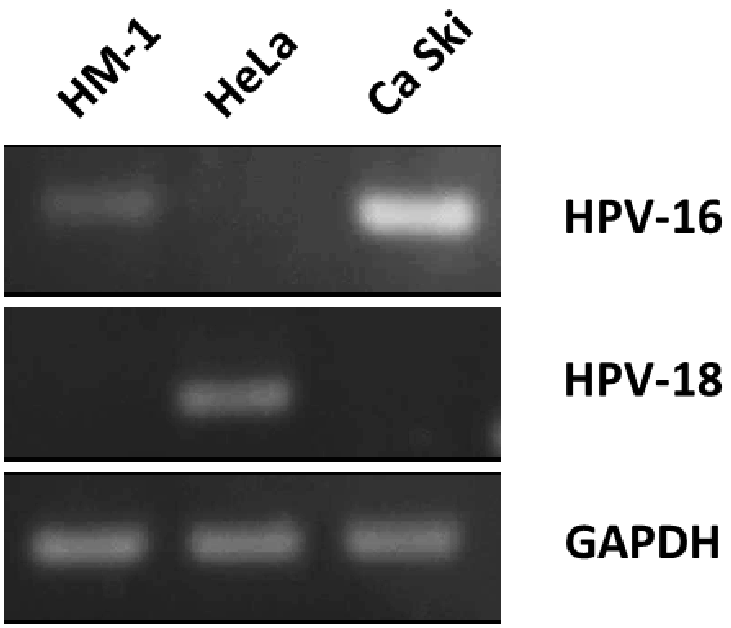

Supplementary Figure 1: Characterization of HM-1 cells. Human papillomavirus (HPV) were detected in HM-1 cells by RT-PCR. The data showed HM-1 cells and Ca Ski cells displayed HPV-16, while HeLa cells expressed HPV-18. Ca Ski (human cervical squamous cell carcinoma cell line) was a HPV-16 control. HeLa (human cervical adenocarcinoma cell line) was a HPV-18 control. GAPDH was a loading control.

Supplementary Table 1: Allele table for the HM-1 cells

STR Locus	Repeat Numbers
D5S818	11, 12
D13S317	8, 12
D7S820	9, 12
D16S539	10, 11
vWA	14, 17
TH01	7, 9
Amelogenin	x
TPOX	8, 11
CSF1PO	11, 12
D21S11	29, 32.2

The short tandem repeat (STR) analysis of HM-1 was carried out at the markers D5S818, D13S317, D7S820, D16S539, vWA, TH01, Amelogenin, TPOX, CSF1PO and D21S11.
